# Balancing demands and resources toward better flourishing and life satisfaction: a two-wave path analysis of school burnout and teens’ resilience

**DOI:** 10.3389/fpsyg.2026.1897438

**Published:** 2026-09-15

**Authors:** Clément Métais, Marie Oger, Fabien Fenouillet, Charles Martin-Krumm

**Affiliations:** 1 CIAMS, University Paris-Saclay, Orsay, France; 2 APCoSS Lab, IFEPSA-UCO, Angers, France; 3 LINP2-2APS, University of Paris Nanterre, Nanterre, France; 4 VCR Team, School of Practicing Psychologists of the Catholic Institute of Paris, Research Unit « Religion, Culture and Society » (EA 7403), Paris, France; 5 INSPIIRE, INSERM, UMR 1319, University of Lorraine, Metz, France

**Keywords:** adolescents, flourishing, perceived demands, perceived resources, resilience, school-burnout

## Abstract

**Introduction:**

Teens’ anxiety-related issues and more generally adolescents’ mental health problems are a growing global concern. These extend to the school domain, where adolescents can suffer from school-burnout. The school-adapted version of the Job Demands-Resources model (JD-R), incorporated with the construct of resilience, might provide with an explanatory framework in regard this field of health. Our first aim was to investigate the predictive relations between all of the variables displayed in the model – that is, the role of optimism, perceived demands and resources, on school burnout and resilience and, in turn, the impact of these latter on flourishing and life satisfaction. Our second aim was to confirm the adequacy of such an alternative framework (i.e., the school-adapted version of the JD-R integrating the resilience construct and some of its known features).

**Methods:**

In total, 525 middle school and high school French students participated in the study which followed a two-wave prospective longitudinal and observational design. Participants were aged from 12 to 18 years old (Mean=14.7 years; SD= 1.55) and included 50.9% of girls (*n* = 267) and 49% of boys (*n* = 257).

**Results:**

Path analysis shows hypotheses were supported and that the model as a whole fits the data correctly, with fit indices (RMSEA= 0.061; SRMR= 0.026; CI 90% [.022–.103]), chi-square goodness-of-fit test (*χ*^2^ (4) = 11.72, *p* < 0.05) and incremental indices (CFI= 0.993; TLI= 0.968) proving to be satisfactory. Perceived demands were positively related to school burnout and alternatively resources and optimism (known resilience feature) were positively related to resilience which, in turn, positively predicted flourishing and life satisfaction. Indirect mediating effects further supported these direct effects.

**Discussion:**

Findings underscore the importance for schools to put a greater emphasis on promoting student resources to foster resilience, reduce burnout, and enhance overall well-being.

## 1 Introduction

Adaptation skills are key components for teens to cope with increasing mental health issues in order for them to engage into adaptative processes and to improve their mental health (Herbert, 2016; Métais et al., 2024; Ungar et al., 2014; Waters, 2014). In addition, academic demands are, among other things, a significant source of stress for adolescents, potentially leading to academic burnout (Lebert-Charron et al., 2021; Meylan et al., 2015; Salmela-Aro and Upadyaya, 2014). With the goal to deepen understandings of essential adaptation processes, this paper will test the adequacy of an innovative layout of the school-adapted version of the “Job Demands-Resources” (JD-R) model (Oger et al., 2022a; Salmela-Aro and Upadyaya, 2014), incorporating the construct of resilience along with some of its major features (Métais et al., 2022).

### 1.1 Teens mental health and school burnout

Adolescents’ mental health is a growing global concern, with rising anxiety-related psychological distress (McCarthy, 2019; McGorry et al., 2022; Tian et al., 2025). Reports from the World Health Organization (WHO) identify mental illness as a major health issue for adolescents, particularly with teens suffering from depression from 15 to 19 years (World Health Organization, 2022, 2023, 2025), a trend exacerbated by the COVID-19 pandemic (Bell et al., 2023; Guo et al., 2020; Khan et al., 2020; Newlove-Delgado et al., 2023; Wolf and Schmitz, 2023; Xiang et al., 2020). In Europe, geographical location where this study was conducted, depression rates are highest among 17–19-year-olds in the UK, followed by 11–16-year-olds (Watson, 2020), while suicide is the second leading cause of mortality among French youth aged 15–24 (Observatoire National du Suicide, 2022).

These mental health challenges extend to the school environment, where adolescents face stress and school burnout (Salmela-Aro and Upadyaya, 2014, 2020; Vansoeterstede et al., 2023). School is a central compound in adolescents’ lives, impacting them physically (e.g., daily attendance), socially (e.g., peer interactions and social media pressures), and mentally (e.g., academic performance expectations). Social media even amplifies pressures related to self- and body-image, contributing to eating disorders, anxiety, and stress (Machillot, 2017; Singh et al., 2024; Société canadienne de pédiatrie, groupe de travail sur la santé numérique, 2019; Vannucci and Ohannessian, 2018). Simultaneously, societal emphasis on educational success and school performance can trigger fear, anxiety, and stress-related illnesses (Bano and Subba, 2025; Cashman et al., 2024; Lebert-Charron et al., 2021; Meylan et al., 2015).

School burnout, defined as exhaustion from an imbalance between school demands and perceived resources (Oger et al., 2022c; Salmela-Aro and Upadyaya, 2014), is a critical outcome of these pressures. In a French cohort (*n* = 468) of students from grades 7 to 12, 54% (*n* = 251) reported high perceived school-related demands (e.g., cognitive or emotional strain from academic work, sometimes within condensed timeframes), 9% (*n* = 40) felt they lacked sufficient socio-emotional resources, and 45% (*n* = 211) scored only average in terms of these perceived resources (Oger et al., 2022c). These perceptions predicted burnout, which in turn leads to poor life satisfaction (Oger et al., 2022a). Such findings highlight that school burnout and related stressors put adolescents at risk of academic failure and dropout but also expose them to detrimental psychological outcomes, including poor life satisfaction (Salmela-Aro and Upadyaya, 2020; Vansoeterstede et al., 2024).

Despite this, the interplay between perceived school demands, resources, and their impact on adolescents’ mental health and life satisfaction remains understudied, particularly in longitudinal contexts.

### 1.2 JD-R model and its school-adapted version

The models by Salmela-Aro and Upadyaya (2014) and Oger et al. (2022a) introduced a school-adapted version of the JD-R established by Demerouti et al. (2001). To help students deal with the challenges putting them at-risk for mental health problems, it is crucial to both learn more about the causes leading them to school burnout and poor mental health, but also about the brighter pathways within which they engage into resilience or other adaptative processes that allows them to stay on top of their mental health. In this regard, a model based on these school-adapted versions of the JD-R (Oger et al., 2022a; Salmela-Aro and Upadyaya, 2014) was built as they allow to investigate to what extent the balance between demands from the school and resources perceived by the students leads either toward burnout or toward more adaptative consequences and better life satisfaction.

As its name implies, the original JD-R model is inherent to the concepts of resources and demands (related to the work environment or to the school environment in the case of its school-adapted version) and it posits that one may be at high-risk of burnout in cases of an imbalance between the two in favor of the demands he or she has to confront (Demerouti et al., 2001). Demands could be defined as the strains related to the psychological, physical, and social/organizational efforts implied in situations and tasks at work, whereas resources are defined as positive/supportive features or assets the individual can access in order to achieve work goals, to reduce the impact of work demands, and to stimulate personal growth (Bakker et al., 2014; Demerouti et al., 2001; Schaufeli and Bakker, 2004). Overall, the model assumes that in situations where the level of resources does not meet the level or amount of demands, individuals might find themselves unable to withstand workloads and will be prone to exhaustion, burnout and, in turn, diminished mental health. On the other hand, resources that equal or surpass the strains associated with the demands will mitigate exhaustion and individuals will better be able to engage in work tasks, leading to improved satisfaction and better psychological health (Demerouti et al., 2001). Resources have been proved to counter deleterious effects of demands, and to favor performance and work engagement when encountering heavy demands (Bakker and Demerouti, 2007).

In both of their studies Salmela-Aro and Upadyaya (2014) and Oger et al. (2022a) operationalized a school-adapted version of the JD-R model in order to explore students’ adaptation to their school environment by investigating the predictive links between demands and resources, and the outcomes either in terms of burnout (and in turn, diminished mental health) or rather in terms of brighter consequences such as better school engagement and improved life satisfaction. Although one study was implemented upon a four-wave longitudinal design over a two-year span (Salmela-Aro and Upadyaya, 2014), while the other covered a period of one school term (Oger et al., 2022a), both were essential, respectively, in order to pave the way as to the adequacy of the JD-R model applied to the school domain and in order to confirm this school applicability of the model in a global SARS-Cov-2 context where teens mental health was negatively affected (Bell et al., 2023).

### 1.3 Resilience and its interplay with the JD-R model

When reflecting about coping with demands from school and engaging into adaptative processes in the face of stressors, to prevent from school burnout and to foster psychological health, one may think of the concept of resilience to have an interplay here. A recent study even put forward that resilience mediates the relationship between basic psychological needs and engagement (Gao et al., 2025), engagement and school burnout being known negative covariates (Oger et al., 2022a; Salmela-Aro and Upadyaya, 2014). Additionally, although looking toward teachers and not students, the interplay between resilience, self-efficacy and wellbeing should here be noted, with Wang et al. (2024) showing resilience to mediate the relationship between self-efficacy and teachers’ wellbeing.

Yet, more importantly, the duality illustrated by the *adversity dimension* and to the *positive adaptation dimension* inherent to the construct of resilience (Fletcher and Sarkar, 2013; Luthar et al., 2000, 2015; Masten, 2014; Wright et al., 2013), reflecting the duality of *demands* and *resources* in the JD-R model, along with the rise of alarming observations about teens psychological health, altogether advocate for resilience to interfere with the JD-R model. Based on this and the knowledge so far, this study brings this interplay to life in an adapted framework.

In psychology, resilience can be referred to as the processes by which a dynamic system, here an individual at a given stage of his or her development and in a given environmental setting, adapts and adjusts successfully to adversity or known threats to adaptation and to system function (Masten, 2014; Métais et al., 2022; Yates et al., 2015). While early research into resilience conceptualized it is as an inborn gift (Anthony, 1974; Pines, 1975) or a fixed trait (Block and Block, 1980) contemporary researchers see resilience as a dynamic state that is shaped by the interaction of the individual with its environment. The cognitive approach to resilience (i.e., a focus on how internal states and cognition shape one’s reaction to the environment), which shaped much of the research in the 1980s–2000s, has then been expanded to a more ecological approach that recognizes external influences. Furthermore, as an illustration of the more recent steps of resilience research, Métais et al. (2022) argue that now resilience conceptualizations have even progressively turned to transactional and constructivist influences which move beyond a subjective eye (i.e., the cognitive approach) and a contextual eye, to rather focus first on the alchemy resulting from a unique situation (given one’s strengths and weaknesses, but also given one’s momentary leaning, state of mind, stage of life and at given time, circumstance, context, culture,…) thus granting the full consideration of resilience with a more situational lens and as a function of the “individual in an environment and [more particularly] their agency” (p. 112). Overall, resilience could be considered as an opposite path to the more unfavorable path reflecting an inability to withstand workloads and demands that lead to progressive exhaustion and burnout.

Yet in regard to its dynamic nature, it should be highlighted that resilience measuring tools struggle to capture the full longitudinal processes of positive adaptation and, therefore, resilience is never truly measurable directly (Luthar et al., 2015). Viewed from a hierarchical perspective, as a concept that includes various subcomponents, resilience is instead often inferred from assessments of an individual’s competencies and resources in terms of (1) their competence or achievements related to developmental stages or other accomplishments, or (2) their social skills, which are demonstrated through secure attachments (see Ainsworth, 1978; Demogeot and Lighezzolo-Alnot, 2014) and positive connections with family, peers, and educators (Luthar et al., 2015).

### 1.4 From antecedents to consequences of resilience: optimism and flourishing

Approaching resilience as a process implies to account for antecedents influencing this process (i.e., internal and external protective factors; Luthar et al., 2000; Prince-Embury, 2014; Wright et al., 2013), as well as consequences resulting from it (i.e., affects and positive effects on the individual). Additionally, when invoking resources in the face of demands within the school-adapted version of the JD-R model, one may think of resources available in the school environment, yet individuals, here students, also possess their own resources that may be of help in dealing with the demands, in mitigating risks for burnout and in engaging into adaptative processes (Masten, 2007; Métais et al., 2022). One such resource, major antecedent of resilience, is optimism (Bonanno and Diminich, 2013; Galatzer-Levy et al., 2018; Liu et al., 2018; Martin-Krumm, 2023; Masten, 2018). Traditionally, optimism relates to believing that outcomes of future events will turn out positively and, more generally it relates to expecting that the future will be positive (Carver and Scheier, 2023). Optimism has often been shown to predict flourishing and better life satisfaction (Bailey et al., 2007; Kardas et al., 2019; Peterson and Chang, 2003; Sharma and Mohan, 2021; Yildirim, 2020). Furthermore, when looking at optimism and pessimism in a more indirect way, within the prospect of explanatory style (Martin-Krumm, 2023; Martin-Krumm et al., 2003; Peterson, 2000), pessimistic individuals regularly attribute bad experiences or situations to causes that are stable in time (i.e., unlikely to ever be different), global in effect (i.e., affecting everything the individual will undertake), and internal (i.e., due to the individual’s own fault) and contrastingly they attribute good events to unstable, specific, and external causes. On the other hand, optimistic individuals approach what happens in their lives with an opposite attributional style (Martin-Krumm, 2023; Peterson, 2000; Peterson and Vaidya, 2001). In this more indirect approach, the positioning of individuals toward optimism or pessimism is to be considered as per their willingness to mobilize resources to achieve set goals and their confidence and beliefs in the means they are equipped with in order to face obstacles and achieve such goals (Martin-Krumm, 2023; Peterson, 2000). These prospects also echo the motivational and effort-driven processes assumed by the JD-R model (Demerouti et al., 2001; Salmela-Aro and Upadyaya, 2014).

As for the consequences related to the different variables evoked in the previous paragraphs or, more generally, the outcomes resulting from these global frameworks, models, and mechanisms, all illustrating processes of adaptation, they all seem to point in the same direction. On the one hand, optimism has been proved to contribute to better coping capacities, to foster wellbeing, and to fare better in general (Kardas et al., 2019; Peterson and Park, 1998; Segerstrom et al., 2017; Yildirim, 2020). On the other, models and processes such as the JD-R model and resilience, when being successful, result in better engagement in tasks, improved life satisfaction, greater wellbeing and psychological health, as well as personal growth (Aburn et al., 2016; Ayed et al., 2019; Buhs and Rudasill, 2016; Demerouti et al., 2001; Schaufeli and Bakker, 2004; Sharma and Mohan, 2021). Previous studies (Oger et al., 2022a,b,c) having examined outcomes of the processes implied in the JD-R model in the form of life satisfaction (i.e., cognitive appraisal of one’s life as it is subjectively perceived to be; Fenouillet et al., 2015), it could now appear essential to incorporate an outcome variable such as wellbeing, or perhaps a component more directly representing wellbeing.

As it has increasingly been proposed to describe wellbeing in a more systematic and multidimensional way (Diener et al., 2010; Huppert and So, 2013; Marsh et al., 2020), the construct of flourishing may express such a component. It has been approved that if wellbeing was only measured by life satisfaction, valuable insights would be left unaccounted (Huppert and So, 2013). Flourishing alleviates this flaw by being referred to as the experience of life going well, in a more complete and multidimensional perspective, combining hedonic (i.e., feeling good, subjective wellbeing) and eudaimonic (i.e., functioning well, psychological wellbeing) aspects of wellbeing (Huppert, 2009; Huppert and So, 2013; Villieux et al., 2016). Along with optimism, flourishing will here settle an alternative framework of the JD-R in the school domain already incorporating the constructs of optimism and resilience (see Figure 1 for an operationalized illustration of the model).

**Figure 1:**
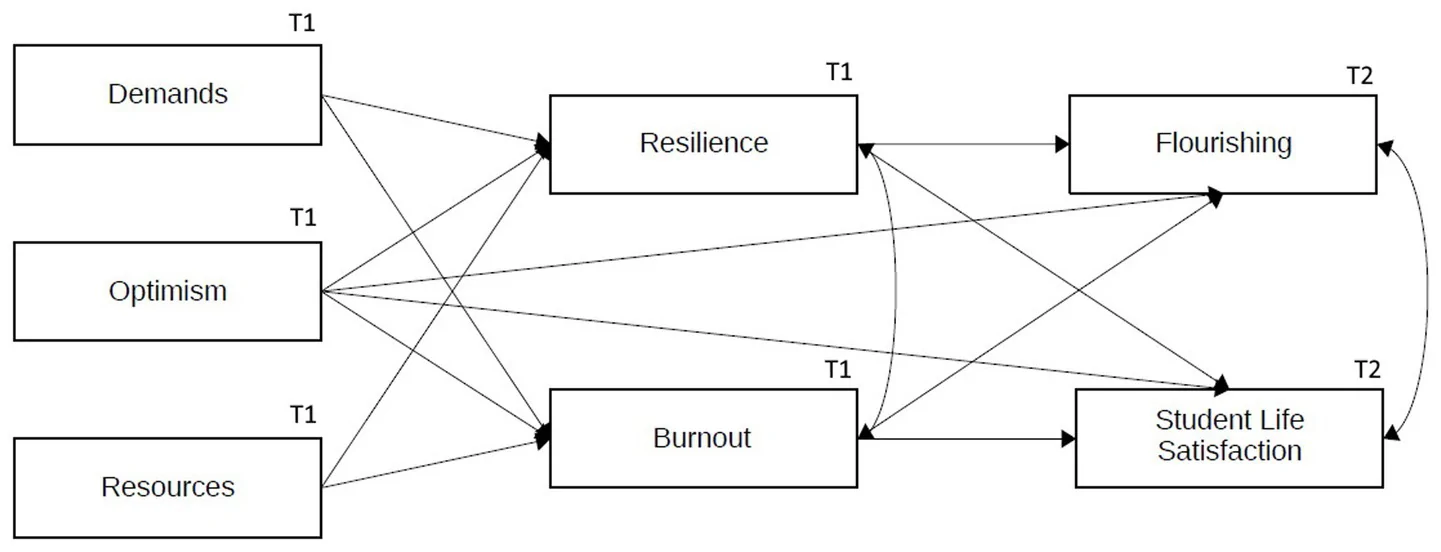
Hypothesized interactional model.

### 1.5 Aims and hypotheses

The goal of this study is to introduce an alternative framework of the school-adapted version of the JD-R model, inspired from studies by Salmela-Aro and Upadyaya (2014) and Oger et al. (2022a). Conjointly, a parallel objective is to confirm the adequacy of such school-adapted approaches to the original JD-R model.

As such a time frame was not explored in the previous studies (Oger et al., 2022a; Salmela-Aro and Upadyaya, 2014), this study was programmed over the course of one school year, beginning at the end of the Autumn term known to be demanding and prone to end-of-year energy drops. More precisely, the primary aim is to investigate the predictive relations between all of the variables displayed in the interactive model (see Figure 1) in a two-time measurement design. The secondary aim is to confirm the adequacy of such an alternative framework (i.e., incorporating the resilience construct and some of its known features) of the school-adapted version of the JD-R model inspired by former studies, in a novel post-COVID global pandemic context. Following findings from the initial JD-R model and from its previous school-adapted versions (Bakker et al., 2014; Oger et al., 2022a; Salmela-Aro and Upadyaya, 2014; Schaufeli and Taris, 2014) the study hypothesizes resources to negatively predict burnout and demands to positively predict it. Upon similar theoretical grounds, the study presumes resources to positively predict resilience and demands to negatively predict it. Moreover, optimism (i.e., personal resource in the JD-R model and antecedent of resilience) is presumed to positively predict resilience and to negatively predict burnout, in accordance with previous work on resilience (for review see Métais et al., 2022) and on school-adapted versions of the JD-R model (Oger et al., 2022a; Salmela-Aro and Upadyaya, 2014). On another level, the study hypothesizes resilience and burnout to act as mediators between the variables advocating for antecedents (i.e., demands, resources, optimism) and the outcome variables (i.e., flourishing, life satisfaction). In the end, it should be noted that no relations were considered between the exogenous variables (i.e., demands, optimism, and resources), as in the studies by Salmela-Aro and Upadyaya (2014) and Oger et al. (2022a).

## 2 Method

### 2.1 Participants

At first, 756 participants were targeted, yet a 30% attrition rate was observed (e.g., students being sick the day of the second measurement time, absent for other reasons, or not registered in the school anymore). Overall, participants included in the data set were 525 in total and were aged 14.7 years old in average (SD = 1.55; Min = 12; Max = 18). They were composed of 50.9% of girls (*n* = 267) and 49% of boys (*n* = 257), whereas one student reported as transgender. They were all French students from 11 schools in the Grand-Est and Nouvelle-Aquitaine French regions (8th grade to 12th grade US) where the corresponding author was able to ask several schools for participation thanks to his network—that is, first circle teacher colleagues and further circles (i.e., colleagues of colleagues). Although it cannot fully solve it, the fact the sample was spread over several different schools and school levels may partially address some school-level clustering biases.

In terms of type of schools, middle schools were general public schools and high schools were either general high schools or vocational agricultural high schools, yet one school was an experimental high school (i.e., mixing vocational and general education and within which teachers run the administrative center, although the school remains accredited by the state like other general schools). As no at-risk population was targeted, the only remaining exclusion criteria was related to a lack of means or lack of will on the teachers’ end—that is, teachers informing us they were not sure they would be able to perform the full study at all measure points were removed from the study and no risk was taken as teachers were the ones to be in charge of administrating the questionnaires to their own students. Response rate was very satisfactory as only two teachers feared not being able to perform all measure points during the whole school year and were therefore removed prior to the study, in the early buildup stages. Note that 11 students (2.1%) were 12 years old at T1 and although this study meant to target 13-year-olds to 18-year-olds (adolescents), these younger students were kept in the sample as they were in their 13th year at the time of the study (i.e., they all turned 13 in December following T1).

In France, it is quite common to find three types of boarding status. Half-boarders (HB) stay all day long at school before going back home in the evening and they have lunch at the school restaurant in between morning classes and afternoon classes. Day students (DS) are going home for lunch break or they have lunch outside of the school and they go home every evening. Full boarders (FB) live all week long at school and they have all their meals at school. This kind of boarding status is nearly always available to families at the high school level only and, in this status, students go home during the weekends and school holidays. An overview of this school boarding status by gender and type of schools is available here below in Table 1.

**Table 1:** Descriptive data according to the school boarding status, gender, and the type of schools.

Boarding status	Gender		Type of schools						Total
			MS	HS	GTHS	VC	ExpHS	Inst	
HB	Gender	F	40	11	15	5	13	27	
		M	31	3	14	4	13	29	
		TR	0	0	0	0	1	0	
	Total		71	14	29	9	27	56	206
DS	Gender	F	77	21	0	0	8	6	
		M	68	19	0	1	1	5	
		TR	0	0	0	0	0	0	
	Total		145	40	0	1	9	11	206
FB	Gender	F	0	0	32	12	0	0	
		M	0	0	41	27	1	0	
		TR	0	0	0	0	0	0	
	Total		0	0	73	39	1	0	113
Total	Gender	F	117	32	47	17	21	33	
		M	99	22	55	32	15	34	
		TR	0	0	0	0	1	0	
	Total		216	54	102	49	37	67	525

HB, half boarder, having lunch at school in between morning classes and afternoon classes (boarding status quite common in France); DS, day students going home for lunch break or having lunch outside of the school; FB, full boarder, having meals and living at school all week-long; TR, identifies as transgender; MS, middle school; HS, standard high school; GTHS, general and technological high school; VC, vocational school; ExpHS, experimental high school which aims at developing self-reliance and sense of responsibility; Inst, institute that does middle school up to high school.

### 2.2 Procedure

This study followed a two-wave prospective longitudinal and observational design. After having collected the study approval from the headmasters of all the schools involved, the students and their parents received a presentation form also serving as a formal authorization form so that they were given the opportunity to discuss at home the choice to participate to the research experiment or not (a typical families’ authorization form is available on demand from the first author). This way students involved in the study were all volunteers and received authorization from their parents.

Measuring scales were administered by the teachers in charge in a paper-and-pencil format during the teachers’ own subject class period. Although it was not always easy for the teachers in charge to set a dedicated time aside for the administration of the questionnaires, they were kindly asked to do so over a two-week window at each of the measure times. Anonymization of the questionnaires was then obtained by giving students a code to report on the front page of the questionnaires (a preview of this front page, displaying the fields for anonymization coding, is also available on demand from the first author). This code was built upon both the first two letters of their family name and the first two letters of their first name, followed by their date of birth (i.e., the precise date they were born, not the month they were born).

As mentioned in earlier studies (Oger et al., 2022a; Salmela-Aro and Upadyaya, 2014), predictive links between the different variables operationalized in a school-adapted version of the JD-R model have not yet been explored over the course of one school year. To this end, the survey took place from the end of the first term (T1; late November/early December 2021) to the end of the third term at the end of the school year (T2; mid/late June 2022). These periods and this time frame were chosen in order to investigate how students could fare and face adversities at a challenging period (i.e., end of the first term)—usually filled with school examinations and prone to exhaustion or end-of-year energy drops—and how this would result in terms of life satisfaction and flourishing at the end of the school year.

Yet the COVID-19 pandemic and its associated restrictions in France during the spring of 2021 caused uncertainty and hesitation among school leaders and administrators about implementing a scientific study in their schools. Consequently, study authorization and operationalization procedures had to be deferred until after the summer break of 2021.

However, with the easing of restrictions at the start of the following academic year, those authorization and operationalization procedures were able to be finalized. Ultimate hierarchical approvals from the school leaders and authorizations from students and parents were obtained in November 2021, hence the inability to fit two different measure time points before the Christmas holidays to space out measures of demands, resources and optimism from measures of resilience and school burnout. In fact, the survey was then conducted at the end of the first trimester (T1; December 2021), as soon as it was possible, straight after those approvals were obtained. When such a setback was foreseen, during the buildup of the study, the question was raised as to whether to cancel the study or to continue—especially based on studies by Salmela-Aro and Upadyaya (2014) and Oger et al. (2022a) who spaced out the measures of these variables as mentioned above. However, two reasons furthered the study and led us to combine the measurement of demands, resources, optimism, resilience, and school burnout into one measure time (i.e., T1) -- T2 being in June (life satisfaction and flourishing measures). First reason, Demerouti et al. (2001) applied JD-R to the workplace, where one measure time was applied for measuring demands, resources, and burnout. Second, the study combined the levels of resilience, and also resources, demands, and burnout, was worth the investigation as these levels would give an assessment of those variables precisely at a time of the year prone to student exhaustion or end-of-year energy drops, as mentioned earlier (e.g., school examinations, pressions regarding end-of-trimester grades, decrease of daylight and lack of sun, etc.).

### 2.3 Measures

A total of six scales were used to measure the variables of interest. Demands and resources, optimism, resilience, and burnout were measured at T1, while flourishing and life satisfaction were measured at T2. Psychometric properties of the scales were all satisfactory. A recap table of the descriptive statistics and psychometric properties of the variables is available as a Supplementary material.

#### 2.3.1 Demands and resources

This study utilized the French scale called “Echelle de Mesure des Exigences et des Ressources à l’école” (EMERE; Oger et al., 2022b). The scale stems from the Conservation of Resources (COR) theory—stating that the stress triggered by adversity is rather due to the objective loss of resources than due to the subjective appraisals of the adversity (Hobfoll et al., 2007; Johnson et al., 2007)—and from the JD-R model (Demerouti et al., 2001) which stipulates that resources may diminish the impact of potentially stressful external demands (e.g., job demands, or perhaps school demands). Upon a two-factor design, the scale allows us to determine the level of resources and demands (requirements) perceived by students at school. It is based on Van de Ven et al.’s (2008) scale, a scale that originally analyses similar components yet in the work domain. The school version used here contains 6 items (3 items for the demands dimension and 3 items for the resources dimension) about which students are asked to indicate to what frequency they found themselves in these situations or feel this way (from “1 = strongly disagree” to “7 = strongly agree”). Scores are to be examined by dimension, the higher the scores are for each of the two dimensions, the higher the perceived demands and the higher the perceived resources. Construct validity of this tool proved to be satisfactory (CFI = 0.99; TLI = 0.99; SRMR = 0.045; RMSEA = 0.06; Oger et al., 2022b), as well as internal consistencies in the dataset of this study, with Cronbach’s alpha (Cronbach, 1951) and omega coefficient (Raykov, 2001), respectively, being at values of 0.68 and 0.72 for demands but also for resources (exact same values).

#### 2.3.2 Optimism

This study used the Life Orientation Test—Revised (LOT-R; Scheier et al., 1994) in which the researchers removed two items with regard to the original scale for those items were more about the adaptative style of the individual rather than about positive expectancies facing the future. The French version of the LOT-R was validated by Trottier et al. (2008). The tool used is a one-factor scale composed of 10 items the subjects were assessed on a 0-to-4 Likert scale (0 = totally disagree; 4 = totally agree). Four of the 10 items are fillers, and 3 of the 6 remaining items are to be inverted. The higher the scores the more optimistic the students are. The scale showed sound construct validity (CFI = 0.98; TLI = n/a; RMSEA = 0.07; Trottier et al., 2008) while the internal consistencies in this study were also satisfactory (α = 0.72 and ω = 0.73).

#### 2.3.3 Resilience

To measure resilience, the study used the CYRM-R, which is a revised version of the Child and Youth Resilience Measure (CYRM-28; Ungar and Liebenberg, 2011). The strength of the original CYRM-28 is that it has a similar approach than the current complex understandings of resilience such as transactional approaches that encompass internal, external, and contextual elements. It is designed as a screening tool to explore the resources available to individuals that may bolster their resilience. By doing so, the CYRM-28 intends to respect the diversity between situations and contexts. As noted in the introduction, however, the CYRM—like other resilience assessment tools—infers a resilience capacity based on individuals’ subjective perception of their competencies and resources, rather than on proven resilience (such capacity having not been tested yet). More precisely, the scale asks students about their competencies or ability to function and cope with various events in their lives, as well as about their positive relationships with peers, family, and other people in their environment.

The scale was created during the International Resilience Project (IRP) organized by the Resilience Research Centre (RRC), a research center gathering many experts in the field of resilience. Translations in many languages of the original and revised versions of the scale are available in open source online on the RRC website (Resilience Research Center, 2024).

The French version of the CYRM-R has a two-factor design (i.e., personal resilience and relational resilience) and the scale contains 17 items (Daigneault et al., 2013; Jefferies et al., 2019). On a 5-point Likert scale, participants are asked to indicate the extent to which they agree with the statements (from “1 = Not at all” to “5 = A lot”). Higher total scores on the scale indicate higher resilience.

In terms of fit indices, TLI and CFI were not indicated in previous validation studies (Daigneault et al., 2013; Jefferies et al., 2019) and, although RMSEA and RMSR fit statistics were good in relation to an initial three-factor solution (respective values of 0.059 and 0.055), the scale was then rash-validated which resulted in a final two-factor design (Jefferies et al., 2019). Additionally, internal consistencies in this study were satisfactory, with Cronbach alpha (Cronbach, 1951) and omega coefficient (Raykov, 2001), respectively, being at values of 0.84 and 0.85.

#### 2.3.4 School burnout

To measure school burnout, the French Athlete Burnout Questionnaire (French ABQ; in French: Échelle de Mesure du Burnout scolaire) was used, validated by Isoard-Gautheur et al. (2010) and based on the athlete burnout questionnaire (Raedeke and Smith, 2001) which has the advantage of being adjustable to several domains and contexts (e.g., sport or school environments). This scale contains 12 items that pertain to 3 fatigue dimensions (i.e., physical/emotional exhaustion, self-devaluation, diminishing of accomplishment feeling). On a 1-to-5 scale, students must indicate the extent to which the 12 statements correspond to what they feel at school (from “1 = Never” to “5 = Always”). The higher the total score on the scale is, the higher the level of burnout is. The scale showed sound construct validity (CFI = 0.96; GFI = 0.95; TLI = n/a; RMSEA = 0.07; Isoard-Gautheur et al., 2010) and the internal consistencies for this study were found to be satisfactory (α = 0.85 and ω = 0.86).

#### 2.3.5 Flourishing

As mentioned earlier, within the past 10 years the term “flourishing” has increasingly been proposed to describe wellbeing in a more systematic way, combining hedonic and eudaimonic dimensions (Diener et al., 2010; Huppert and So, 2013; Marsh et al., 2020). The French version (Villieux et al., 2016) of the Flourishing Scale (FS; Diener et al., 2010) was used in this study. Factor analysis and eigenvalues showed a one-factor structure for the French version to be the best factor solution (Villieux et al., 2016). This version also includes eight items and is assessed on a 7-point Likert scale ranging from 1 (strong disagreement) to 7 (strong agreement). High scores indicate that respondents view themselves positively regarding important areas of functioning or that they have many psychological resources and strengths. The scale showed sound construct validity (CFI = 0.91; TLI = n/a; RMSEA = 0.061; Villieux et al., 2016) and psychometric properties in this study also indicated satisfactory consistency (α = 0.87 and ω = 0.87).

#### 2.3.6 Life satisfaction

Life satisfaction relates to the subjective perception of one’s appraisal of his or her own life. In order to measure the quality of life of the students at school, the study used Huebner’s Multidimensional Students’ Life Satisfaction Scale (Huebner, 1994). The French version of the scale, validated by Fenouillet et al. (2015), is a 5-factor scale (i.e., family, friends, school, living place, self-perception) divided in 30 items that need to be assessed on a 7-point Likert scale ranging from “1 = I do not agree at all” to “7 = I completely agree.” A single total score is to be calculated, with the higher the score the better students are satisfied with life at school. The scale showed sound construct validity (CFI = 0.93; TLI = 0.92; RMSEA = 0.07; Fenouillet et al., 2015) and the Cronbach alpha and omega coefficient also proved the internal consistency in this study to be very satisfactory (α = 0.91 and ω = 0.92).

### 2.4 Data analysis

Jamovi (version 2.3.28), an open source “3rd generation” statistical software, was used to process and analyze the data of the entire experiment. It exhibits a spreadsheet display built upon the R statistical language (R code may be extracted if necessary). After having extracted the descriptive statistics and having performed the reliability analysis, a sample variance–covariance–correlation matrix was edited (see Table 2). Additionally, differences in the variance of burnout and resilience baseline levels based on the boarding status (i.e., HB, DS, FB) were examined with Jamovi using a one-way ANOVA. Yet such preliminary examination revealed no significant differences in the variance of burnout (*η*^2^ = 0.004, *p* = 0.343) and resilience levels (*η*^2^ = 0.024, *p* = 0.002) based on boarding status; therefore, these variables were not included as covariates in the main path analysis to preserve the model’s degrees of freedom. Another one-way ANOVA also showed similar lack of variance of burnout and resilience based on the type of school (i.e., general middle school and high school, vocational agricultural high schools, experimental high school).

**Table 2:** Sample variance–covariance–correlation matrix (*N* = 525).

Variables	*N*	mean	SD	1	2	3	4	5	6	7
1. Optimism T1	525	12.2	4.43	**19.63**	−0.177***	0.314***	0.487***	−0.430***	0.433***	0.438***
2. Demands T1	525	14.5	4.01	−3.14	**16.05**	−0.014	−0.026	0.255***	−0.076	−0.077
3. Resources T1	525	15.9	4.10	5.70	−0.23	**16.80**	0.499***	−0.256***	0.375***	0.394***
4. Resilience T1	525	61.4	9.82	21.20	−1.02	20.08	**96.41**	−0.417***	0.526***	0.575***
5. Burnout T1	525	34.7	9.35	−17.82	9.53	−9.83	−38.29	**87.37**	−0.317***	−0.379***
6. Flourishing T2	525	42.5	7.47	14.33	−2.27	11.47	38.53	−22.14	**55.74**	0.737***
7. Life satisfaction T2	525	150	25.5	49.40	−7.89	41.15	143.84	−90.31	140.23	**648.87**

Variance is displayed in the diagonal; covariance and correlation’s coefficients are, respectively, positioned below and above the diagonal. Asterisks indicate significant Pearson’s correlation coefficients as follows: *p < 0.05; **p < 0.01; ***p < 0.001. The bolded values specified limits of the diagonal.

Path analysis was performed using Mplus 8.11 (Muthén and Muthén, 2017, Muthén et al., 2017). The maximum likelihood estimator (ML) was selected to estimate the model and the standardized coefficients (*β*) of its various paths. Moreover, to evaluate the overall fit of the model, the recommendations of Hu and Bentler (1999) were followed and the study interpreted the goodness-of-fit indices.

Specifically, the study considered absolute indices to be adequate if they were less than 0.06 or 0.08 for the root mean square and the SRMR, respectively. The study also considered incremental indices to be adequate if they were greater than 0.95 for the comparative fit index (CFI) and the Tucker–Lewis index (TLI) (Kline, 2023). To estimate the direct and indirect effects, 95% bias-corrected confidence intervals (CI) were derived based on 1,000 bootstrap samples (Preacher and Hayes, 2008).

## 3 Results

The goodness-of-fit indices show that the model does quite fit the data correctly. In fact, although the upper 90% CI seems a bit high, it could still be suggested that the RMSEA and the SRMR remain satisfactory (RMSEA = 0.061; SRMR = 0.026; CI 90% [0.022–0.103]) as well as the chi-square goodness-of-fit test with *χ*^2^ (4) = 11.72, *p* < 0.05. In terms of incremental indices, CFI and TLI are greater than 0.95 and therefore also show good general adequacy (CFI = 0.993; TLI = 0.968).

Furthermore, as in Figure 2, analysis of the local indices (path analyses and covariances) indicates that the predictive direct paths are globally significant. More precisely, demands (*β* = 0.19; 95% CI [0.11, 0.26]), resources (*β* = −0.14; 95% CI [−0.22, −0.06]) as well as optimism (*β* = −0.35; 95% CI [−0.43, −0.26]) at T1 predict burnout at the same time of the school year, which in turn negatively predicts life satisfaction (*β* = −0.12; 95% CI [−0.21, −0.04]) at the end of the school year (T2), yet fails to negatively predict flourishing (*β* = −0.06; 95% CI [−0.15, 0.03]). Similarly, resources (*β* = 0.38; 95% CI [0.31, 0.46]) and optimism (*β* = 0.38; 95% CI [0.30, 0.44]) at T1, but not demands (*β* = 0.05; 95% CI [−0.02, 0.12]), predict resilience at the same time of the year, which in turn positively predicts both flourishing (*β* = 0.40; 95% CI [0.31, 0.48]) and life satisfaction (*β* = 0.44; 95% CI [0.36, 0.52]) at T2. Additionally, significant covariances are observed between resilience and burnout (Cov = −0.25; 95% CI [−0.33, −0.16]) and between flourishing and life satisfaction (Cov = 0.60; 95% CI [0.53, 0.67]).

**Figure 2:**
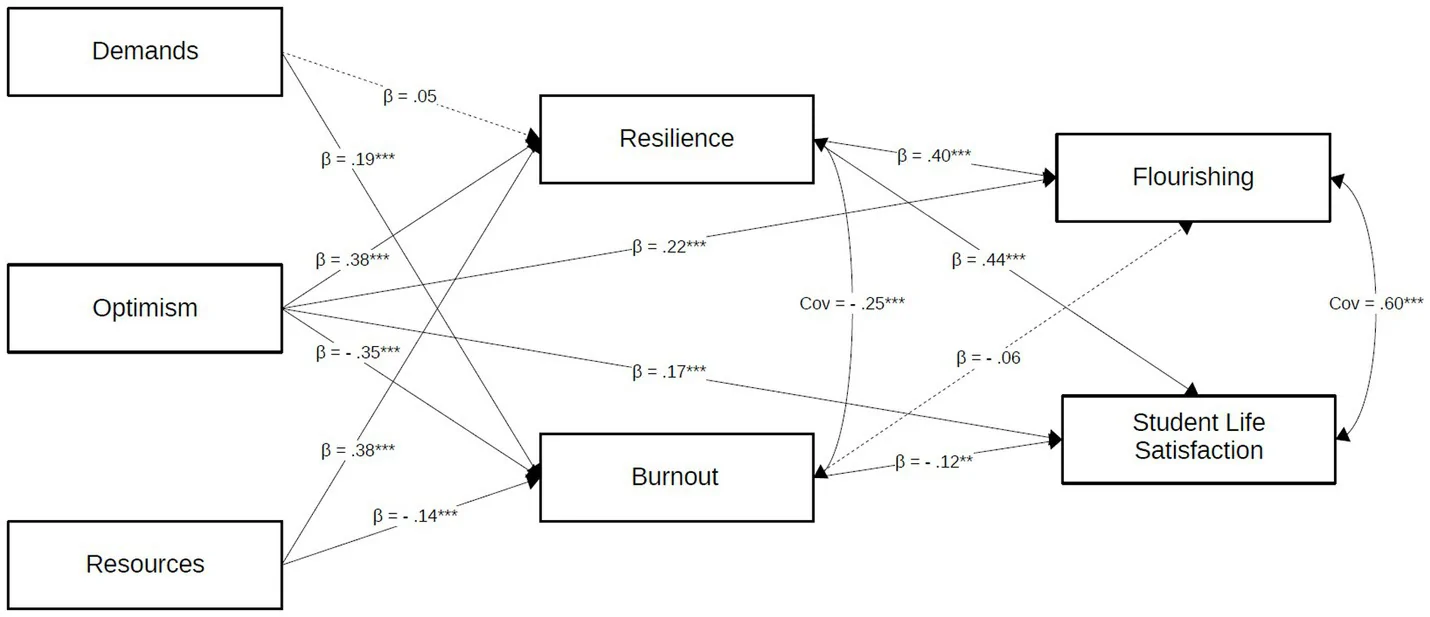
Analysis of predictive paths between variables of interest. **p < 0.01; ***p < 0.001.

Regarding indirect effects, the effect of optimism on flourishing is mediated by resilience (*β* = 0.15; 95% CI [0.11, 0.20]) but not by burnout (*β* = 0.02; 95% CI [−0.01, 0.06]). The effect of demand on flourishing is mediated neither by resilience (*β* = 0.02; 95% CI [−0.01, 0.05]) nor by burnout (*β* = −0.01; 95% CI [−0.03, 0.01]). The effect of resources on flourishing is mediated by resilience (*β* = 0.15; 95% CI [0.11, 0.20]) but not by burnout (*β* = 0.01; 95% CI [−0.004, 0.03]). The effect of optimism on life satisfaction is mediated by resilience (*β* = 0.17; 95% CI [0.12, 0.21]) and burnout (*β* = 0.04; 95% CI [0.01, 0.08]). The effect of demands on life satisfaction is mediated by burnout (*β* = −0.02; 95% CI [−0.05, −0.01]) but not by resilience (*β* = 0.02; 95% CI [−0.01, 0.05]). Finally, the effect of resources on life satisfaction is mediated by resilience (*β* = 0.17; 95% CI [0.12, 0.22]) and burnout (*β* = 0.02; 95% CI [0.003, 0.04]).

## 4 Discussion

This study and the design of the school-adapted version of the JD-R model, in which was incorporated optimism, resilience, flourishing and life satisfaction, was inspired by studies from Salmela-Aro and Upadyaya (2014) and Oger et al. (2022a). These studies, respectively, acknowledged the adequacy of the demands-resources model in the school domain over a two-year period (i.e., demands and resources fully predicting school engagement and burnout, mediators to life satisfaction or depressive symptoms) and predictive links between the variables over the course of one school term (i.e., resources predicting burnout and engagement, demands predicting burnout which in turn, along with engagement, predicted satisfaction or negative adaptation).

As for the present study, the primary aim was to investigate the predictive relations between all of the variables displayed in the interactive model incorporating the JD-R model—the constructs of optimism, resilience, and flourishing. For a second time this study intended to bring a contribution to the existing literature rather than to test a specificity in priority, so that the second aim was to confirm the adequacy of the school-adapted version of the JD-R model incorporating resilience and some of its features, in a post pandemic context. But more importantly, as for the current times where teens’ mental health becomes more and more concerning worldwide (McGorry et al., 2022; World Health Organization, 2022), this study was more generally motivated by the urge to identify and understand the mechanisms, intricacies, and potential interplays between features involved in or illustrating adaptation processes, which in turn may help teens mitigate stress and enhance their wellbeing.

### 4.1 Main findings

This study followed students along the school year, according to a design comprised of two measurement times going from the end of the first school term to the end of the third and final term of the school year. This aspect may be considered a major strength of this study as it allowed to examine the predictive relations between the variables of interest on a temporal frame never tested before. Additionally, it should be noted that this study showed satisfactory statistical power in that more than 500 participants were involved.

Considering such design, this study has managed to successfully incorporate the school-adapted version of the JD-R model the construct of resilience, along with some of its related features. The results reveal direct predictive links between demands-resources and burnout, in turn negatively predicting life satisfaction. Such direct effects go hand in hand with the indirect effects showing that both perceived demands and resources act on life satisfaction through the mediating role of burnout. These results make it clear that students’ perception of the resources available and of the demands they face—and more specifically the balance between both (i.e., do the levels of perceived resources meet, or even surpass, the levels of perceived demands?)—will either trigger or prevent from burnout and, overall, will influence life satisfaction (either by altering it or by promoting it). These align with and reinforce the results of Salmela-Aro and Upadyaya (2014) and Oger et al. (2022a), yet in a school context post COVID global pandemic, depicted by deteriorating teens’ mental health. Furthermore, results of this study also bring novel evidence about the adequacy of a school-adapted JD-R model incorporated with the construct of resilience along with optimism (i.e., antecedent, personal resource) and flourishing (i.e., consequence of resilience), as they show resources and optimism to have a direct effect on resilience, which in turn positively predicts flourishing and life satisfaction. Here again, these direct effects go conjointly with the indirect effects as to perceived resources and optimism were showed to act on both flourishing and life satisfaction through the mediating role of resilience. Such findings support the idea of resilience along with some of its core features having to play within a school-adapted JD-R model such as the model assumed earlier (see Figure 1). In short, perception from the students of the resources available (i.e., school resources and personal resources such as optimism) plays a crucial role as it positively affects their mental health (i.e., flourishing and life satisfaction) through the mediating role of burnout or enhancing it through the mediating role of resilience.

A potential reason for the absence of a connection between demands and resilience is that the employed resilience tool does not incorporate aspects linked to adversity. As mentioned earlier when reflecting about the inability for measuring tools to capture the dynamic nature of resilience, such a construct is almost systematically inferred from assessing an individual’s abilities and resources (Luthar et al., 2015), such as (1) their competence or success in tasks related to developmental milestones or other achievements, or (2) social competencies operationalized through attachments (see Ainsworth, 1978; Demogeot and Lighezzolo-Alnot, 2014) and positive relationships with family, peers, and teachers (Luthar et al., 2015). The CYRM-R tool in question (Daigneault et al., 2013; Jefferies et al., 2019) follows this approach, focusing on these positive skills and relationships rather than including items about demands and adversities.

### 4.2 Implications and perspectives—can schools act on the development of students’ resources and help them face demands and adversity?

Some argue that schools represent ideal places to embed resilience interventions (Masten et al., 2008; McHale and Maidrag, 2015; Ungar et al., 2014) that can have large-scale delivery and, thus wide-scale impact on the mental health prevention of teenagers (Challen et al., 2014; Stallard and Buck, 2013). Schools have also been identified by the WHO as key institutions to help young people cope with the global pandemic (World Health Organization, 2020). Capurso et al. (2020) assert that it is important for schools to help children build their resilience and sense of meaning in order to process the critical events connected to the COVID pandemic. Several other authors have also called for the need for schools to build “resilient systems” around young people during the COVID global pandemic (Masten and Motti-Stefanidi, 2020).

A notable implication is first conceivable regarding the fact that (1) demands, resources, and optimism have a direct effect on student burnout, (2) resources and optimism (but not demands) have a direct effect on resilience which, in turn (3) has a mediating role on both flourishing and life satisfaction. Whether to be attained through the implementation of a school-based resilience program, or simply through choices made by the school in terms of areas to target, these findings touch on a potential balance to be found between promoting student wellbeing and resources and reducing their distress. The lack of correlation between demands and resilience, while the association between resources and resilience is satisfactory (the latter also predicting flourishing and life satisfaction), clearly indicates that schools or school resilience programs could place an even greater emphasis on promoting student resources. This could be targeted through class time or activities that aim for students to discover their character strengths and the range of human strengths or skills that exist (Boniwell and Martin-Krumm, 2014; Oger and Martin-Krumm, submitted). Personal strengths positively influencing wellbeing and resilience (Harzer, 2016; Martínez-Martí and Ruch, 2017), conditions could be created within the school for the students to apply their strengths or develop new ones, such as organizing events where the students experience positive emotions, both through leisure activities and activities where they put their strengths to use and feel useful. Then external resources could also be facilitated by establishing a strong culture of positive relationships and positive interactions (e.g., active-constructive responding; see Gable et al., 2004).

Moreover, the positive link between optimism and resilience, which in turn fosters flourishing, also suggests that schools could focus on this relationship. For instance, under the supervision of school psychologists, schools could introduce teacher–student (or student-to-student) discussion sessions in order for students to explore how they explain adversity or their tendencies to interpret and explain negative events. Optimism, defined as a relatively stable anticipatory state or more generally a positive expectation about future events (Alarcon et al., 2013; Carver et al., 2010; Carver and Scheier, 2023; Martin-Krumm, 2023), may be challenging to cultivate or modify due to its stability. However, through these discussion sessions, schools could give students a supportive environment to explore and question explanations they attribute to positive or negative events—aligning with the concept of explanatory style (Peterson and Buchanan, 1995)—and, when needed, develop alternative narratives.

### 4.3 Limitations

Several limitations must nonetheless be acknowledged. While the model’s fit indices were satisfactory in the initial study, two correlations were weak and non-significant (specifically, between demands and resilience, and between exhaustion and flourishing), suggesting that the model did not perform sufficiently well compared to the null model (Kenny, 2020). Despite this, the satisfactory results for the rest of the model allowed for some conceptual interpretations. Additionally, several parameters not explored in this study could be investigated in future research. These include the differential impact of age or gender on the variables of interest, using methods such as multi-group confirmatory factor analysis (Jöreskog, 1971), as well as variations in correlations based on different dimensions of the variables of interest (e.g., the two dimensions of the resilience tool—personal resilience and relational resilience; the five dimensions of the life satisfaction tool). Future studies could also examine the impact of demands and resources on resilience at multiple points during the school year, and the impact of resilience on flourishing throughout the school year using a longitudinal design.

Other potential limitations pertain to the methods used in this study. First, the reliance on self-report measures may introduce common method variance (CMV; Podsakoff et al., 2003). Closely related to CMV, self-report measures—that are already quite subjective assessments—may also bring some bias as to the results may reflect the respondents’ mood and social desirability, rather than objective results. To address these limitations, future research could incorporate multi-method approaches, such as behavioral observations or objective data. Second, although one of the interests was to investigate a joint picture of the levels of resilience, but also resources, demands and burnout, such design implies a concurrent mediation, as both the independent variable and the mediator were assessed at the same time point. This may preclude strong causal inferences about the mediating process. Future research should employ even more of a longitudinal (more than the two-wave design) or experimental design to establish temporal precedence and strengthen causal claims. Third and final, the use of convenience sampling, as schools and classes were recruited through the first author’s professional network, may have introduced selection bias. Participating schools could differ systematically from non-participating ones (e.g., in terms of resources, motivation of the teachers in charge of administrating the questionnaires, or student demographics). As a result, the generalizability of the findings to the broader population of schools may be limited. Future research should aim to use random sampling to ensure a more representative sample.

## 5 Conclusion

Globally, the results of this study confirm previous findings that school demands and resources predict, respectively, positively and negatively school burnout (Oger et al., 2022a,c; Salmela-Aro and Upadyaya, 2014). Additionally, optimism, resilience, flourishing, and life satisfaction were successfully incorporated in the model as to the results of this study revealing resources and optimism to positively predict resilience which in turn positively predicts flourishing and life satisfaction. Furthermore, these results suggest resilience to mitigate school burnout. All in all, although schools can represent a demanding environment for students in terms of rules to be respected, workload, and success expectancies, they may also bear the conditions for them to access resources (e.g., social support from peers and adults) and to develop or increase their own personal resources (e.g., character strengths). As per the global alarming picture of teens mental health, such goals seem crucial to consider and implement in schools. Life satisfaction and student wellbeing is a marker that should be considered on par with academic success and end-of-year school results.

## Data Availability

The original contributions presented in the study are included in the article/supplementary material, further inquiries can be directed to the corresponding author.
